# Continuous Antibiotic Prophylaxis for Vesicoureteral Reflux: Impact on the Pediatric Microbiome—A Systematic Review

**DOI:** 10.3390/children12111446

**Published:** 2025-10-24

**Authors:** Olivia Oana Stanciu, Andreea Moga, Laura Balanescu, Radu Balanescu, Mircea Andriescu

**Affiliations:** 1 Department of Pediatric Surgery and Orthopedics, “Carol Davila” University of Medicine and Pharmacy, 050474 Bucharest, Romania; olivia.stanciu@drd.umfcd.ro (O.O.S.); laura.balanescu@umfcd.ro (L.B.); radu.balanescu@umfcd.ro (R.B.); mircea.andriescu@umfcd.ro (M.A.); 2 Pediatric Surgery Department, “Grigore Alexandrescu” Clinical Emergency Hospital for Children, 011743 Bucharest, Romania

**Keywords:** vesicoureteral reflux, antibiotic prophylaxis, microbiome, urinary tract infection, infant, pediatric urology

## Abstract

**Highlights:**

**What are the main findings?**

- Continuous antibiotic prophylaxis (CAP) in children with vesicoureteral reflux preserves overall gut microbial diversity but induces subtle compositional shifts, including enrichment of *Enterobacteriaceae* and reduction in *Bifidobacteriaceae*.
- Functional analyses show that the core fermentative capacity of the gut microbiota (e.g., short-chain fatty acid production) remains stable under CAP, although the resistome may expand with prolonged exposure.

**What are the implications of the main findings?**

- Even mild antibiotic-driven dysbiosis during infancy may influence immune and metabolic development, underscoring the need for microbiome-aware prophylaxis strategies.
- Identifying microbiome alterations could guide personalized interventions—such as optimized antibiotic regimens, shorter prophylaxis duration, or probiotic supplementation—to preserve microbial health while preventing urinary tract infections.

**Abstract:**

**Background**: Continuous antibiotic prophylaxis (CAP) is widely used in infants with vesicoureteral reflux (VUR) to prevent recurrent urinary tract infections and renal scarring. However, this practice entails prolonged low-dose antibiotic exposure during a critical period of microbiome establishment, potentially influencing long-term microbial and immune development. **Methods**: A systematic review was conducted according to PRISMA 2020 guidelines. PubMed, Embase, Scopus, Web of Science, and the Cochrane Library were searched up to September 2025 for studies evaluating gut or urinary microbiome changes in children receiving CAP for VUR. Eligible studies included human participants under 18 years with microbiome outcomes assessed by sequencing or culture-based methods. **Results**: Twenty-one records were identified, and four studies met inclusion criteria—three observational microbiome studies and one randomized controlled trial. CAP preserved overall microbial alpha diversity but induced compositional changes, notably enrichment of *Enterobacteriaceae* and reduction in Bifidobacteriaceae. The included RCT confirmed reduced UTI recurrence but increased antimicrobial resistance and non–*E. coli* infections. **Conclusions**: CAP in early life maintains microbial diversity but alters microbiota composition and resistance profiles. Identifying these shifts may support individualized prophylaxis strategies and microbiome-preserving interventions to balance infection prevention with ecological safety in infancy.

## 1. Introduction

Vesicoureteral reflux (VUR) is a prevalent congenital urinary tract anomaly characterized by the retrograde flow of urine from the bladder into the ureters and kidneys. It affects approximately 1–3% of children, with a higher prevalence among those presenting with febrile urinary tract infections (UTIs) [1]. Continuous antibiotic prophylaxis (CAP) has been the cornerstone of VUR management for decades, aimed at reducing recurrent UTIs and preventing renal scarring. Typical regimens include low-dose trimethoprim-sulfamethoxazole, nitrofurantoin, or cephalexin administered daily over extended periods, often months or years.

While the Randomized Intervention for Children with Vesicoureteral Reflux (RIVUR) trial and subsequent studies demonstrated modest efficacy of CAP in preventing recurrent infections, growing concern surrounds its broader biological impact—particularly on the developing pediatric microbiome [2]. The early-life period is critical for microbiome establishment, which plays a fundamental role in immune maturation, metabolic programming, and resistance to pathogens. Antibiotic exposure during this sensitive window can disrupt microbial community composition, decrease diversity, and promote colonization with antibiotic-resistant organisms [3].

Emerging evidence indicates that even short courses of antibiotics can cause measurable and sometimes persistent alterations in gut and urinary microbiota [3,4]. Continuous or long-term prophylaxis, by design, entails sustained exposure that may amplify these effects. Such perturbations could have clinical consequences, including increased risk of allergic disease, obesity, and future infections with resistant strains.

Continuous antibiotic prophylaxis (CAP) is widely used in infants with vesicoureteral reflux (VUR) with/without recurrent urinary tract infections to prevent renal damage. Most recipients are under one year of age, exposed to low-dose antibiotics during a critical period of microbiome development. Emerging evidence shows that CAP can alter microbial composition—reducing *Bifidobacteriaceae* and enriching *Enterobacteriaceae*—even when overall diversity is preserved. Identifying these shifts may enable targeted interventions, such as probiotic support or shorter prophylactic regimens, to maintain microbial balance while preserving CAP’s protective effect.

Despite increasing recognition of the microbiome’s importance in pediatric health, no prior systematic review has specifically examined the effects of continuous antibiotic prophylaxis (CAP) for vesicoureteral reflux (VUR) on both the gut and bladder microbiota. Existing reviews have focused broadly on antibiotic exposure during infancy or early childhood rather than on long-term, low-dose prophylactic regimens. For example, Luchen et al. (2023) evaluated antibiotic-associated changes in gut microbiome diversity and resistome development in infants from low- and middle-income countries, while Wurm et al. (2024) synthesized evidence on the intestinal effects of various antibiotic classes in children [3,5]. However, these reviews did not distinguish between prophylactic and therapeutic antibiotic use, nor did they assess urinary microbiome outcomes. The present study therefore addresses a critical gap by systematically reviewing CAP for VUR, with specific attention to both gut and bladder microbiome alterations in the pediatric population.

This review aims to determine how continuous antibiotic prophylaxis for vesicoureteral reflux affects the gut and bladder microbiome of children.

## 2. Methods

This systematic review follows the Preferred Reporting Items for Systematic Reviews and Meta-Analyses (PRISMA) 2020 guidelines and was prospectively registered in the PROSPERO database (https://www.crd.york.ac.uk/PROSPERO/view/CRD420251161209, accessed on 5 October 2025).

### 2.1. Eligibility Criteria

Population: Children aged 0–18 years with a diagnosis of vesicoureteral reflux confirmed radiologically (any grade).

Intervention: Continuous antibiotic prophylaxis (CAP) administered for the prevention of recurrent urinary tract infections, regardless of drug type, dose, or duration (minimum duration ≥ 1 month).

Comparator: Placebo, no prophylaxis, or pre/post-intervention comparisons within the same cohort.

Outcomes:

Primary outcomes: Changes in gut and/or urinary microbiome composition assessed by culture-independent methods (e.g., 16S rRNA sequencing, shotgun metagenomics); diversity and richness indices (Shannon, Simpson, Chao1); relative abundance of major bacterial taxa; detection of antimicrobial resistance genes or resistant organisms.

Secondary outcomes: association between microbiome alterations and clinical outcomes (e.g., recurrent UTI, colonization resistance).

Study Design: Randomized controlled trials (RCTs), cohort studies, case–control studies, and before–after studies reporting microbiome outcomes.

Exclusion Criteria: Studies in adults (>18 years); animal or in vitro studies; case reports, editorials, reviews, or commentaries; studies lacking microbiome data.

### 2.2. Information Sources and Search Strategy

A systematic search was conducted across five major bibliographic databases—PubMed/MEDLINE, Embase (Ovid, Providence, RI, USA), Scopus (Elsevier, Amsterdam, The Netherlands), Web of Science (Clarivate, Philadelphia, PA, USA), and the Cochrane Library (CENTRAL, Mumbai, India)—to identify studies evaluating the impact of continuous antibiotic prophylaxis (CAP) for vesicoureteral reflux (VUR) on the pediatric microbiome. The search strategy was developed in accordance with the PRISMA 2020 guidelines and combined controlled vocabulary (e.g., MeSH, Emtree) and free-text terms related to vesicoureteral reflux, antibiotic prophylaxis, continuous, and microbiome or microbiota.

The final database search was completed on 15 September 2025.

Search terms were adapted for each database, using Boolean operators and truncations as appropriate. The detailed strategies for each database are available in Table 1. Searches were limited to English-language studies involving human participants. No restrictions were applied to publication date or study design.

Clinical trial registers (including ClinicalTrials.gov, WHO International Clinical Trials Registry Platform [ICTRP], EU Clinical Trials Register, and ISRCTN) were screened informally, but no additional ongoing or unpublished studies meeting inclusion criteria were identified; therefore, these sources were not included in the quantitative search set.

Reference lists of all included papers and relevant reviews were screened to identify additional studies.

### 2.3. Study Selection

**Figure 1:**
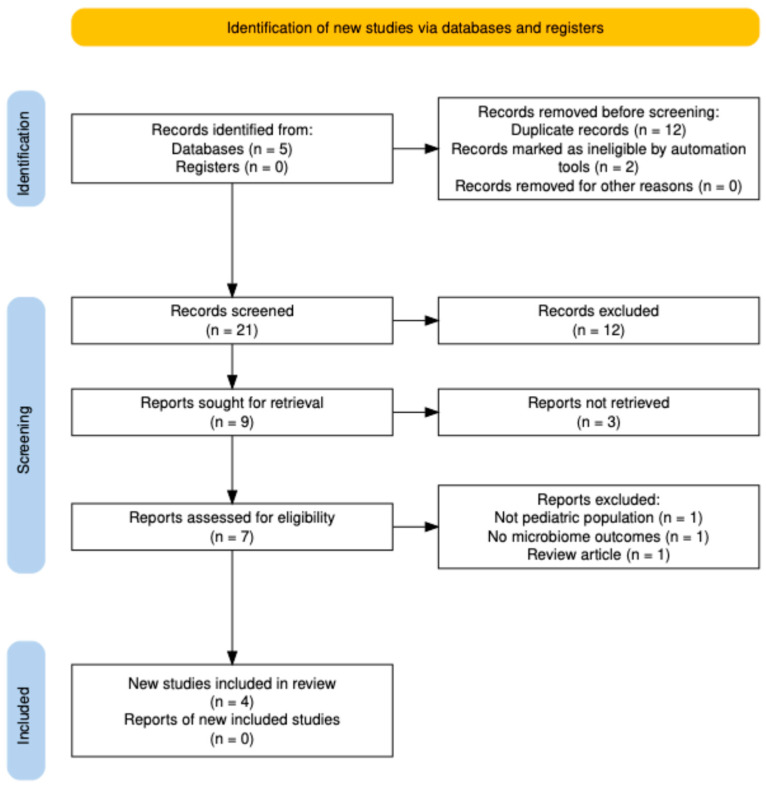
PRISMA 2020 flow diagram showing the selection process for studies evaluating continuous antibiotic prophylaxis and the pediatric microbiome. A total of 21 records were identified, 12 duplicates removed, and 7 full-text articles assessed; 4 studies were included in the qualitative synthesis.

Two reviewers independently screened titles and abstracts using predefined inclusion and exclusion criteria. Full texts were retrieved for studies meeting or potentially meeting the criteria. Discrepancies were resolved by consensus or consultation with a third reviewer. The study selection process is summarized in a PRISMA flow diagram (Figure 1).

### 2.4. Data Extraction

A standardized data extraction form was developed to collect study characteristics (author, year, country, design); participant demographics and VUR grade; type, dose, and duration of antibiotic prophylaxis; microbiome sampling site (gut, urinary), timing, and sequencing method; alpha and beta diversity metrics; taxonomic and functional changes; reported antibiotic resistance data. Data was extracted independently by two reviewers, and discrepancies resolved by discussion.

### 2.5. Risk of Bias Assessment

Risk of bias was assessed using appropriate tools according to study design: randomized trials: Cochrane Risk of Bias 2 (RoB 2); observational studies: Newcastle–Ottawa Scale (NOS); before–after designs: Joanna Briggs Institute (JBI) checklist. Each domain was judged as low, high, or unclear risk of bias.

### 2.6. Data Synthesis

Given the expected heterogeneity in study design, antibiotic types, and microbiome analysis methods, a narrative synthesis was the primary approach.

If ≥3 studies report comparable quantitative measures (e.g., alpha diversity indices), a random-effects meta-analysis was conducted using appropriate statistical software.

Findings are presented in summary tables stratified by antibiotic class (e.g., trimethoprim-sulfamethoxazole, nitrofurantoin); microbiome site (gut vs. urinary); study design and population characteristics.

### 2.7. Certainty of Evidence

The GRADE (Grading of Recommendations Assessment, Development, and Evaluation) approach was used to assess the overall certainty of evidence for each outcome, classifying it as high, moderate, low, or very low.

## 3. Results

### 3.1. Study Selection

The combined database search yielded 21 records. After removal of 12 duplicates, 9 unique records remained. Two records were excluded automatically (non-research or outside scope), leaving 7 titles and abstracts for manual screening. All seven articles were assessed in full text, of which three were excluded for the following reasons: non-pediatric population (*n* = 1), no microbiome outcome (*n* = 1), and narrative review (*n* = 1). A total of four studies met the inclusion criteria and were incorporated into the qualitative synthesis: three observational studies that directly analyzed the pediatric gut microbiome during CAP, and one randomized controlled trial (PREDICT, *NEJM*, 2023 [6]) evaluating the clinical and resistance outcomes of long-term CAP in infants with high-grade VUR. No studies were eligible for quantitative meta-analysis because of heterogeneity in microbiome sequencing methods and outcome reporting. The study selection process is summarized in Figure 1 (PRISMA 2020 flow diagram).

### 3.2. Study Characteristics

The four included studies were published between 2020 and 2023 and enrolled a total of 426 children (infants to early childhood) (Table 2).

Designs comprised one multicenter RCT (*PREDICT*, NEJM 2023 [6]) and three prospective observational or cross-sectional studies (*Morello 2021* [7], *Strasser 2020* [8], *Akagawa 2020* [9]).

Sample sizes ranged from 12 to 292 participants.

Antibiotics included trimethoprim–sulfamethoxazole (TMP-SMX) and cefaclor, administered continuously for weeks to months (median duration 47 days to 24 months).

Three studies assessed gut microbiota by 16S rRNA gene sequencing of stool samples, while the RCT analyzed urine cultures and resistance patterns as ecological outcomes.

### 3.3. Quality and Risk of Bias

The overall methodological quality was moderate (Table 3). The single RCT (PREDICT) demonstrated low risk of bias across domains. Observational studies varied in sample size and lacked blinding, yielding moderate risk per Newcastle–Ottawa criteria. No study performed metagenomic or resistome profiling. No study performed randomization or adjustment for potential confounders such as diet, breastfeeding status, or prior antibiotic exposure.

### 3.4. Impact on Gut Microbiome Composition

#### 3.4.1. Alpha and Beta Diversity

Across studies, alpha diversity (richness and evenness of microbial taxa) was generally preserved under continuous antibiotic prophylaxis. Morello et al., 2021 [7] and Strasser et al., 2020 [8] reported no significant differences in alpha diversity between CAP and control groups. Akagawa et al., 2020 [9] observed a transient decline in diversity during acute infection treatment that normalized during CAP, suggesting microbiome resilience. Morello 2021 [7] detected significant beta-diversity separation (weighted UniFrac *p* = 0.015) between CAP and no-CAP infants, indicating compositional shifts despite stable overall richness. Strasser 2020 [8] and Akagawa 2020 [9] showed no significant between-group separation.

#### 3.4.2. Taxonomic Shifts

Notable compositional alterations included: increased relative abundance of *Enterobacteriaceae*, *Bacteroidaceae*, and *Parabacteroidaceae*; decreased *Bifidobacteriaceae* levels after CAP exposure; TMP-SMX CAP was associated with lower proportions of *Enterobacteriaceae* compared with no prophylaxis (Akagawa et al., 2020 [9]).

#### 3.4.3. Functional and Metabolic Findings

Two studies (Morello 2021 [7]; Strasser 2020 [8]) analyzed short-chain fatty acids (SCFAs) and branched-chain fatty acids (BCFAs), showing no significant metabolic alterations between CAP and controls. No included study performed metagenomic or resistome profiling.

### 3.5. Clinical and Resistance Outcomes from the PREDICT Trial (NEJM 2023 [6])

The multicenter PREDICT RCT (39 European centers; *n* = 292 infants, 80% grade IV–V VUR) directly compared CAP vs. no CAP over 24 months.

CAP reduced the first UTI risk (hazard ratio 0.55; 95% CI 0.35–0.86; number needed to treat ≈ 7) but led to higher rates of antimicrobial resistance and a shift from *E. coli* to non–*E. coli* pathogens in breakthrough infections.

Although the trial did not perform microbiome sequencing, these findings demonstrate that long-term CAP exerts measurable selective pressures within the host and urinary ecosystem.

### 3.6. Synthesis of Findings

When integrated across the four studies: CAP preserves microbial richness (alpha diversity) but modestly alters community structure (beta diversity) in some infant cohorts; taxonomic shifts point toward *Enterobacteriaceae* enrichment and reduced *Bifidobacteriaceae*, consistent with low-dose antibiotic selective pressure; the PREDICT trial confirms a clinically significant benefit in UTI prevention balanced by greater resistance and pathogen replacement.

Together, these data suggest that CAP’s microbiological impact is subtle at the microbiome level yet measurable at the pathogen ecology level, highlighting the need for longitudinal multi-omic investigation linking microbial perturbation with clinical outcomes.

## 4. Discussion

This systematic review identified four studies evaluating the impact of continuous antibiotic prophylaxis on the pediatric microbiome.

Despite diverse methodologies, findings consistently indicate minimal changes in microbial diversity but detectable compositional shifts, particularly involving *Enterobacteriaceae* expansion and loss of beneficial taxa such as *Bifidobacteriaceae*.

Evidence remains limited, with short follow-up durations, small sample sizes, and lack of functional analyses.

### 4.1. Microbiome Diversity and Composition

All sequencing-based studies (Morello et al., 2021 [7]; Strasser et al., 2020 [8]; Akagawa et al., 2020 [9]) demonstrated that α-diversity (species richness and evenness) remained stable during CAP. This suggests that low-dose, long-term antibiotic exposure does not cause gross depletion of the gut microbial community in otherwise healthy infants [4,10].

However, β-diversity analyses revealed modest but reproducible shifts in microbial composition, reflecting selective pressures rather than global disruption. CAP cohorts showed relative enrichment of *Enterobacteriaceae*, *Bacteroidaceae*, and *Parabacteroidaceae*, alongside decreased *Bifidobacteriaceae*, a genus associated with mucosal health and early immune maturation [3,11]. Such taxonomic trends are consistent with mild antibiotic-driven dysbiosis and may predispose to altered metabolic or immune responses later in childhood.

### 4.2. Biological Interpretation

Continuous exposure to low-dose antibiotics may exert subtle yet sustained selective pressure on the developing microbiome. While alpha diversity often appears preserved, this stability may mask underlying compositional shifts, including the enrichment of *Enterobacteriaceae*, *Bacteroidaceae*, and *Parabacteroidaceae* alongside the depletion of beneficial taxa such as *Bifidobacteriaceae* and *Lactobacillaceae* [7,8,9]. These alterations reflect selective suppression of antibiotic-sensitive commensals and compensatory expansion of resistant or opportunistic species. In early life, even minor taxonomic imbalances can influence immune education, mucosal barrier development, and metabolic programming, potentially predisposing to later allergic, metabolic, or inflammatory disorders [3,11,12].

The apparent preservation of overall diversity may reflect the intrinsic resilience of the infant microbiome, particularly among breastfed infants, whose milk-derived oligosaccharides selectively nourish *Bifidobacteriaceae* and support recolonization following antibiotic exposure [13]. Antibiotic-specific effects also contribute—narrow-spectrum agents such as nitrofurantoin may cause limited collateral damage, whereas trimethoprim–sulfamethoxazole or cephalosporins exert broader ecological disruption [14]. Persistent *Enterobacteriaceae* enrichment is of special concern, as these taxa frequently harbor transferable resistance genes, serving as reservoirs for antimicrobial resistance and reducing colonization resistance against pathogens [15]. Collectively, these findings suggest that continuous antibiotic prophylaxis in infancy imposes ecological selection rather than overt microbial depletion, warranting careful antibiotic choice, shorter prophylactic courses, and microbiome monitoring to mitigate long-term risks.

### 4.3. Functional and Metabolic Outcomes

Two included studies quantified fecal short-chain fatty acids (SCFAs)—the principal metabolic products of microbial fermentation of dietary fibers—as well as branched-chain fatty acids (BCFAs) derived from protein metabolism, finding no significant differences in concentration profiles between children receiving continuous antibiotic prophylaxis (CAP) and untreated controls [7,8,9]. These results suggest that the core fermentative and metabolic capacity of the gut microbiota remains largely preserved under low-dose, long-term antibiotic exposure, at least over short- to medium-term follow-up (6 weeks to 3 months). Maintenance of total SCFA output indicates that functionally redundant microbial taxa may compensate for antibiotic-sensitive species, thereby sustaining metabolic stability despite compositional shifts [15].

However, subtle changes in relative SCFA ratios—such as reduced butyrate-producing *Faecalibacterium* or *Roseburia* spp.—have been reported in pediatric antibiotic exposure studies, potentially influencing epithelial integrity, mucosal immunity, and regulatory T-cell differentiation [3]. Thus, while CAP does not appear to cause major metabolic collapse, even modest alterations in microbial function during early life could influence immune and metabolic programming with long-term clinical implications.

The ecological impact of continuous antibiotic prophylaxis likely varies according to the pharmacologic spectrum and intestinal bioavailability of the agent used. Trimethoprim–sulfamethoxazole (TMP-SMX), the most frequently prescribed prophylactic drug, exhibits limited anaerobic activity and moderate gut exposure, potentially explaining the preservation of overall diversity observed in several studies [9,16]. In contrast, broader-spectrum or anaerobe-active antibiotics—such as amoxicillin/clavulanate or second-generation cephalosporins—exert more extensive effects on commensal taxa, particularly *Bifidobacteriaceae* and *Lactobacillaceae*, leading to greater disruption of gut community structure and increased selection for resistant organisms. Cephalosporins like cefaclor, though less systemically absorbed, may still alter microbial composition through biliary excretion and selective pressure on facultative anaerobes [17]. These pharmacologic differences underscore the importance of antibiotic stewardship in prophylactic protocols: whenever possible, agents with narrow spectra, limited intestinal activity, and short systemic half-lives should be prioritized to minimize ecological disturbance while maintaining clinical efficacy.

### 4.4. Clinical Implications

While CAP remains effective in preventing recurrent UTI, clinicians must weigh microbiome perturbation and antimicrobial resistance risks against its benefits. Potential strategies include periodic reassessment of CAP necessity, narrow-spectrum agent selection, adjunctive probiotics or microbiome-supportive measures, and future development of non-antibiotic prophylaxis options.

The PREDICT trial (NEJM 2023) [6] adds critical evidence linking microbiological stability with clinical risk-benefit balance. CAP significantly reduced the risk of recurrent urinary tract infection (hazard ratio 0.55; 95% CI 0.35–0.86), confirming prophylactic efficacy in high-grade VUR. However, it also increased the prevalence of antibiotic-resistant organisms and shifted breakthrough infections from *E. coli* toward non-*E. coli* uropathogens [6]. Although the study did not include microbiome sequencing, these ecological findings reinforce that CAP exerts measurable selective pressures within host and urinary microbial ecosystems.

Continuous antibiotic prophylaxis (CAP) remains widely prescribed in pediatric urology, particularly during infancy. Children under one year of age constitute the largest group receiving low-dose CAP, often for vesicoureteral reflux or recurrent urinary tract infections, due to their high susceptibility to infection and limited ability to verbalize symptoms [6]. Although the preventive rationale is well established, prolonged daily exposure to trimethoprim-sulfamethoxazole, cephalexin, or nitrofurantoin occurs during a critical period of microbiome development [6,8,9]. Identifying the specific microbial alterations associated with CAP—such as relative enrichment of *Enterobacteriaceae* and reduction in beneficial genera like *Bifidobacteriaceae*—may enable early microbiological monitoring and the design of corrective strategies [3,11,15]. These could include modulation of antibiotic choice or duration, probiotic supplementation, or dietary interventions to support microbiome resilience. A precision-microbiome approach may thus optimize infection prevention while mitigating ecological disruption in the youngest and most vulnerable patients [12,13].

Children with vesicoureteral reflux and recurrent urinary tract infections may already exhibit microbiome alterations prior to antibiotic exposure. Recurrent infections, inflammation, and repeated urinary stasis can modify both the urinary and gut microbial ecosystems, reducing microbial diversity and favoring the dominance of uropathogenic *Enterobacteriaceae* species. Recent studies using culture-independent methods have demonstrated that the pediatric urobiome is not sterile but harbors commensal taxa such as *Lactobacillus*, *Corynebacterium*, and *Streptococcus*, which contribute to urinary tract homeostasis [4,10]. In children with VUR or recurrent UTIs, this balance appears disrupted, with depletion of protective commensals and enrichment of pathogenic genera even before prophylactic therapy begins. Similar trends have been observed in the gut microbiota, where recurrent infections and inflammation are associated with decreased *Bifidobacteriaceae* and increased *Enterobacteriaceae* abundance [18,19]. These baseline dysbiosis patterns suggest that infection-related ecological disturbance may compound the selective pressure exerted by continuous antibiotic prophylaxis, potentially amplifying microbiome shifts and resistance selection.

The observed compositional shifts—namely enrichment of *Enterobacteriaceae* and reduction in *Bifidobacteriaceae*—may carry important clinical implications even in the absence of overt dysbiosis. *Enterobacteriaceae* expansion is associated with reduced colonization resistance and increased susceptibility to antibiotic-associated diarrhea and infection by opportunistic pathogens. Conversely, depletion of *Bifidobacteriaceae*, a keystone genus in early-life immune education, has been linked to impaired gut barrier function, diminished short-chain fatty acid production, and greater risk of allergic sensitization and immune dysregulation. These microbial alterations, though subtle, may therefore contribute to downstream outcomes such as heightened infection risk, inflammatory or metabolic disturbances, and antibiotic resistance propagation. While the current evidence does not establish causality, recognizing these potential links underscores the importance of microbiome-aware clinical strategies—such as probiotic supplementation, dietary modulation, and judicious antibiotic use—to preserve microbial resilience during continuous prophylaxis.

### 4.5. Integrative Interpretation

Together, these data suggest that CAP exerts targeted ecological selection rather than broad microbiome depletion. The absence of major diversity loss may reflect partial microbial resilience, frequent recolonization, or limited systemic exposure from low-dose regimens. Nevertheless, observed compositional changes and resistance emergence raise concerns about long-term ecological consequences—especially during the critical window of immune and metabolic programming in early life. The combined evidence supports a cautious, individualized use of CAP, favoring risk-stratified protocols and periodic reassessment of necessity.

Although evidence directly linking continuous antibiotic prophylaxis to long-term health outcomes in children with vesicoureteral reflux remains limited, insights from other pediatric populations highlight the broader clinical relevance of early-life microbiome disturbance. In cohorts undergoing hematopoietic stem-cell transplantation, for instance, profound antibiotic-driven loss of microbial diversity has been associated with impaired immune reconstitution, increased infection risk, and adverse survival outcomes [20]. Similarly, repeated antibiotic exposure during infancy has been correlated with higher incidence of allergic disease, obesity, and metabolic dysregulation, reflecting the microbiome’s central role in immune and metabolic programming. Extrapolating from these contexts suggests that even the subtle compositional shifts observed under long-term low-dose prophylaxis could have cumulative biological effects over time. Integrating such comparative evidence emphasizes the need for longitudinal, multi-omic studies to clarify whether the microbiome perturbations observed in vesicoureteral reflux prophylaxis translate into measurable clinical consequences.

### 4.6. Comparison with Existing Literature

These results align with broader pediatric antibiotic-microbiome studies demonstrating early-life perturbations following systemic antibiotic use. However, unlike acute high-dose exposures, CAP’s long-term, low-dose regimen may cause subtle, cumulative effects rather than overt dysbiosis. Previous meta-analyses on CAP in VUR (e.g., RIVUR 2014 [2]) focus on clinical efficacy, not microbiological outcomes—reinforcing this review’s novelty.

Findings from this review align partially with previous systematic analyses exploring the effects of antibiotics on the pediatric microbiome, though important distinctions exist in study scope and population. Luchen et al. (2023) reported that repeated antibiotic exposure in infants from low- to middle-income countries led to marked reductions in microbial diversity and enrichment of resistance genes, reflecting the cumulative effects of broad-spectrum treatments [5]. Vallianou et al. (2021) highlighted how long-term antibiotic-induced microbiome alterations may contribute to metabolic dysregulation and obesity risk, emphasizing systemic consequences beyond infection control [21]. Wurm et al. (2024) synthesized pediatric data across multiple antibiotic classes, observing consistent decreases in *Bifidobacteriaceae* and *Lactobacillaceae* regardless of indication or duration [3].

Unlike previous reviews assessing general antibiotic exposure, our analysis focuses exclusively on children undergoing continuous antibiotic prophylaxis for vesicoureteral reflux—a uniquely long-term, low-dose exposure scenario that occurs during a critical period of microbiome development. This focused approach allows for a more precise understanding of how sustained antimicrobial pressure shapes both gut and urinary microbial ecosystems. By synthesizing findings across studies evaluating the gut and, to a lesser extent, the bladder microbiome, this review highlights that while overall microbial diversity is generally preserved, compositional shifts and emerging resistance patterns are evident. The integration of gut and urinary microbiome perspectives provides a more comprehensive view of how continuous prophylaxis influences microbial ecology throughout the pediatric urinary tract, emphasizing the need for microbiome-conscious strategies in the management of vesicoureteral reflux.

### 4.7. Strengths and Limitations of Current Evidence

This review synthesizes both microbial and clinical outcomes, offering an integrated perspective seldom addressed in previous CAP studies. However, available microbiome data are limited by small sample sizes, heterogeneous sequencing methods, and short follow-up durations. None of the included studies performed shotgun metagenomic or resistome profiling, precluding functional inference. Furthermore, all microbiome studies were observational, and potential confounders—such as diet, mode of delivery, and prior antibiotic exposure—were variably controlled.

Future research should employ longitudinal, multi-omic designs combining metagenomics, metabolomics, and culture-based resistance tracking to clarify the mechanistic links between CAP, microbiome resilience, and clinical outcomes.

Limitations include small number of included studies, heterogeneous designs, antibiotics, and sequencing methods, short follow-up periods, lack of standardized microbiome outcomes, inability to perform meta-analysis, and publication bias toward small or positive findings.

This review intentionally limited inclusion to children with vesicoureteral reflux (VUR) to provide a focused evaluation of continuous antibiotic prophylaxis (CAP) in a well-defined clinical context. VUR represents the most common indication for long-term, low-dose antibiotic use in early childhood, allowing for consistent comparison across studies with similar therapeutic goals and exposure durations. Restricting the analysis to this population minimized confounding related to differing infection types or treatment regimens. However, this narrow scope also represents a limitation, as findings may not be fully generalizable to other pediatric conditions requiring prolonged antibiotic use, such as recurrent urinary tract infections without reflux or postsurgical prophylaxis.

### 4.8. Usefulness in Current Practice

This systematic review provides a comprehensive evaluation of the available evidence on how continuous antibiotic prophylaxis (CAP) for vesicoureteral reflux (VUR) influences the pediatric microbiome. By integrating current findings, it offers clinicians a clearer understanding of the balance between the potential benefits of infection prevention and the possible long-term consequences on microbial diversity and resistance development. The review supports more informed, individualized decision-making regarding CAP use in children with VUR and emphasizes the need for careful risk–benefit assessment before initiating prolonged antibiotic regimens. Additionally, it highlights the importance of monitoring microbiome-related outcomes in clinical practice and may contribute to the refinement of future guidelines, promoting antibiotic stewardship and the protection of pediatric microbiome health.

### 4.9. Future Directions

Future research should incorporate longitudinal metagenomic and resistome analyses; evaluate functional outcomes (e.g., SCFAs, host immunity); include larger multicenter pediatric cohorts; explore microbiome restoration strategies following CAP cessation.

Future research should extend beyond vesicoureteral reflux to evaluate the microbiome effects of continuous antibiotic prophylaxis in other pediatric contexts, including recurrent urinary tract infections, neurogenic bladder, or congenital anomalies requiring chronic prophylaxis. Comparative studies examining alternative or adjunctive strategies—such as probiotic supplementation, immunomodulatory therapies, or bladder-targeted interventions—could help identify microbiome-preserving approaches that maintain infection protection while minimizing ecological disruption. Integrating multi-omic analyses, longitudinal follow-up, and functional assessments will be essential to clarify whether microbiome alterations observed during CAP translate into meaningful clinical or developmental outcomes.

## 5. Conclusions

Current evidence suggests that continuous antibiotic prophylaxis for vesicoureteral reflux may preserve overall gut microbial diversity while inducing subtle but measurable compositional shifts, including enrichment of *Enterobacteriaceae* and reduction in beneficial taxa such as *Bifidobacteriaceae*. These changes indicate selective ecological pressure rather than widespread microbiome depletion. Evidence regarding the bladder microbiome remains extremely limited, with most available data derived from culture-based rather than sequencing approaches. Although continuous prophylaxis remains clinically effective in reducing urinary tract infection recurrence, the ecological implications of long-term antibiotic exposure in early life warrant careful consideration. Given the small number of available studies, methodological heterogeneity, and short follow-up durations, the findings of this review should be interpreted with caution. Larger, longitudinal, multi-omic studies are needed to confirm these observations and to determine whether the observed microbial alterations translate into meaningful clinical or developmental consequences for children receiving prolonged antibiotic prophylaxis.

## Figures and Tables

**Table 1:** Full electronic search strategy used for each database. Comprehensive search strategies were developed for each database in alignment with PRISMA 2020 guidelines. The search combined controlled vocabulary terms (e.g., MeSH, Emtree) and free-text keywords related to vesicoureteral reflux, antibiotic prophylaxis, and microbiome outcomes in pediatric populations.

Database/Source	Date Searched	Search Strategy (Boolean/Controlled Vocabulary)	Records Retrieved (*n*)
PubMed/MEDLINE	15 September 2025	(„vesicoureteral reflux“[MeSH Terms] OR „vesicoureteral reflux“[tiab] OR VUR[tiab]) AND („anti-bacterial agents“[MeSH Terms] OR antibiotic*[tiab]) AND (prophylaxis[tiab] OR „continuous“[tiab] OR „low-dose“[tiab] OR „long-term“[tiab]) AND (microbiome[tiab] OR microbiota[tiab] OR „gut flora“[tiab] OR „urinary microbiome“[tiab]) AND (infant*[tiab] OR child*[tiab] OR pediatric*[tiab])	9
Embase (Ovid)	15 September 2025	1. ‚vesicoureteral reflux‘/exp OR (vesicoureter* NEAR/3 reflux): ti, ab 2. ‚antibiotic prophylaxis‘/exp OR (antibiotic* NEAR/3 prophylaxis): ti, ab 3. (continuous OR ‚low dose‘ OR low-dose OR ‚long term‘ OR long-term):ti,ab 4. Microbiome/exp OR microbiota/exp OR (microbiom* OR microbiot* OR ‚gut flora‘):ti,ab 5. Infant/exp OR child/exp OR pediatric/exp OR (infant* OR child* OR pediatric*):ti,ab 6. 1 AND 2 AND 3 AND 4 AND 5 7. Limit 6 to human AND English	5
Scopus (Elsevier)	15 September 2025	TITLE-ABS-KEY(„vesicoureteral reflux“ OR VUR) AND TITLE-ABS-KEY(antibiotic* W/3 prophylaxis) AND TITLE-ABS-KEY(continuous OR „low-dose“ OR „long-term“) AND TITLE-ABS-KEY(microbiome OR microbiota OR „gut flora“ OR „urinary microbiome“) AND TITLE-ABS-KEY(infant* OR child* OR pediatric*) AND (LIMIT-TO(LANGUAGE, „English“))	3
Web of Science (Clarivate)	15 September 2025	TS = („vesicoureteral reflux“ OR VUR) AND TS = (antibiotic* NEAR/3 prophylaxis) AND TS = (continuous OR „low-dose“ OR „long-term“) AND TS = (microbiome OR microbiota OR „gut flora“ OR „urinary microbiome“) AND TS = (infant* OR child* OR pediatric*) AND LANGUAGE: (English)	2
Cochrane Library (CENTRAL)	15 September 2025	(vesicoureter* reflux):ti,ab,kw AND (prophylaxis OR antibiotic*):ti,ab,kw AND (child* OR pediatric* OR infant*):ti,ab,kw	2
Total	—	—	21

* Notes: Search results were exported in RIS and CSV formats and imported into Zotero for deduplication. Twelve duplicates were identified and removed. The final unique record set (*n* = 9) was screened manually. Registers (ClinicalTrials.gov, WHO ICTRP, EU CTR) were not systematically searched, as no ongoing pediatric CAP–microbiome studies were identified. Full search documentation (syntax, date run, export files) is archived with the PROSPERO submission.

**Table 2:** Characteristics of included studies.

Parameter/Study	Morello et al., 2021 [7] Front Pediatr	Strasser et al., 2020 [8] J Pediatr Urol	Akagawa et al., 2020 [9] J Urol	PREDICT Trial, 2023 NEJM [6]
Country/Setting	Italy	Austria	Japan	Multicenter (39 European sites)
Design/Population	Cross-sectional observational study of infants with VUR receiving CAP vs. no CAP	Prospective longitudinal cohort of infants with urogenital malformations on CAP	Prospective cohort of children with VUR on long-term CAP	Randomized controlled trial of infants with grade III–V VUR, CAP vs. no CAP
Sample Size (*n*)	87	12	35	292
Age Range	1–12 months	<1 year	6 months–6 years	1–5 months
Antibiotic Prophylaxis Regimen	TMP-SMX (median 47 days)	Cefaclor (continuous ≈ 70 days)	TMP-SMX (>3 months)	TMP-SMX (24 months continuous)
Comparator/Control	No prophylaxis	Healthy age-matched controls	No prophylaxis	Placebo/no CAP
Microbiome Sample/Analysis	Stool samples; 16S rRNA gene sequencing	Stool samples; 16S rRNA sequencing; SCFA analysis	Stool samples; 16S rRNA sequencing	Urine cultures; resistance profiling (no sequencing)
Alpha Diversity	Not clearly reported as different; composition shifted	No change	No change	Not assessed
Beta Diversity	Significant separation (weighted UniFrac *p* = 0.015)	No significant shift	Minimal between-group difference	Not assessed
Key Taxa Enriched/Depleted	↑ Enterobacteriaceae, Bacteroidaceae; ↓ Bifidobacteriaceae	↑ Enterobacteriaceae (transient); ↓ Bifidobacteriaceae	↓ Enterobacteriaceae; minor compositional variation	↑ Resistant Enterobacteriaceae in urine
Main Findings	CAP associated with compositional shift despite preserved overall diversity; functional stability maintained.	Gut diversity stable; metabolic (SCFA) profiles unchanged during CAP.	α-diversity preserved; limited compositional change over time.	CAP reduced UTI recurrence (HR 0.55) but increased resistant, non-*E. coli* infections.

Abbreviations: CAP = continuous antibiotic prophylaxis; VUR = vesicoureteral reflux; TMP-SMX = trimethoprim–sulfamethoxazole; SCFA = short-chain fatty acid; ↑ = increase; ↓ = decrease.

**Table 3:** Risk of bias and methodological quality of included studies. Summary: Three observational studies were rated moderate quality due to small sample sizes and limited confounder control. The PREDICT RCT was low risk of bias across all domains, yielding an overall moderate-to-high level of evidence.

Study	Design/Tool Used	Selection Bias	Performance Bias	Detection Bias	Attrition Bias	Reporting Bias	Overall Risk of Bias/Quality Rating
Morello et al., 2021 [7] Front Pediatr	Observational cross-sectional/NIH Tool	Low	Moderate (no blinding)	Low	Low	Low	Moderate
Strasser et al., 2020 [8] J Pediatr Urol	Prospective cohort/NIH Tool	Low	Moderate	Low	Low	Low	Moderate
Akagawa et al., 2020 [9] J Urol	Prospective cohort/NIH Tool	Low	Moderate	Low	Low	Low	Moderate
PREDICT Trial, 2023 NEJM [6]	Randomized controlled trial/Cochrane RoB 2.0	Low	Low	Low	Low	Low	Low risk of bias

## Data Availability

No new data were created or analyzed in this study. Data supporting the findings of this systematic review are derived from the previously published studies cited within the article, all of which are available in public repositories (e.g., PubMed, Scopus, Web of Science, Cochrane Library).

## Abbreviations

The following abbreviations are used in this manuscript:

VUR	Vesicoureteral Reflux
UTI	Urinary Tract Infection
TMP-SMX	Trimethoprim-Sulfamethoxazole
CAP	Continuous Antibiotic Prophylaxis
